# Effects of a Video of Science Rejection by a Social Media Influencer and User Comments: Randomized Controlled Trial

**DOI:** 10.2196/79917

**Published:** 2025-11-17

**Authors:** Benedikt Till, Thomas Niederkrotenthaler, Brigitte Naderer

**Affiliations:** 1 Public Mental Health Research Unit, Department of Social and Preventive Medicine Center for Public Health Medical University of Vienna Vienna Austria

**Keywords:** science communication, misinformation, social media influencer, user comments, science rejection

## Abstract

**Background:**

Rejection of science is a common phenomenon on social media, but little is known about the effects of videos by social media influencers who reject basic principles of science and how other users could effectively counter these false claims.

**Objective:**

This study aimed to explore the effects of an online video on social media that openly rejects facts of medical science on users’ attitudes toward science and scientists, as well as to examine whether different response strategies by other users in the commentary section are effective in altering the impact of the video.

**Methods:**

For this experiment, 470 adults were randomized to 1 of 5 groups. Each group watched either a video rejecting science related to medical research or footage unrelated to science. The science rejection video groups were also exposed to critical comments that focused on factual information, personal attacks on the influencer, a mix of these responses, or they received no comments. We collected data on trust in scientists, attitudes toward scientific research, science support, interest in scientific research, belief in science, conspiracy beliefs, and evaluation of video, influencer, and user comments presented to the participants.

**Results:**

Participants rated the video and influencer less favorably in the science rejection conditions than in the control group, and opposing user comments with factual information tended to be perceived as most informative and trustworthy, but there were no other differences between the 5 experimental conditions. However, in an additional exploratory analysis comparing all 4 science rejection groups with the control group, we found a decrease in interest in scientific research and an increase in trust in scientists. This can potentially be attributed to reactance to the science rejection content due to the sample consisting predominantly of individuals with a high affinity toward science.

**Conclusions:**

Portrayals of science rejection in a video on social media had minimal impact on the attitudes toward science and scientists among users with a high affinity for scientific research. There was limited exploratory evidence suggesting that such videos might slightly increase trust in scientists while decreasing interest in research, but this finding requires further study for confirmation. Furthermore, whereas fact-based user comments tended to be considered more informative and trustworthy than other comments, none of the tested response strategies significantly changed the video’s impact, highlighting the need for more research, particularly in individuals with little or no affinity toward scientific research who might be particularly vulnerable to the examined video content.

**Trial Registration:**

German Clinical Trial Registry DRKS00033829; https://drks.de/search/en/trial/DRKS00033829/details

## Introduction

### Background

Science is a fundamental component of modern society that influences many aspects of life, including technology, communication, and health care. Understanding scientific research empowers individuals to make better decisions about their health, environment, and daily lives, and helps prevent the development and strengthening of common misconceptions and myths in society. A basic understanding of medical science is a key component in health literacy, which can even become essential for survival in the face of health crises and is subsequently crucial for social safety [1]. Thus, the systematic and unwarranted rejection or denial of the value of science is a major public health problem that can have serious consequences for both individuals and society [2].

The rejection of science is differentiated from scientific skepticism in the literature on the basis of its more extreme claims or accusations, such as labeling scientists as “corrupt,” “liars,” or members of a “cartel” [3]. The rejection of science can concern specific topics, such as COVID-19, vaccinations, climate change, or evolutionary theory [3-8], but can also comprise science in its entirety as a reliable source of knowledge about the world [9]. Science rejecters or deniers publicly oppose the robust results of scientific inquiry and spread misinformation, resulting in potentially biased public opinions that can affect important societal decisions [10]. We use the term “misinformation” as opposed to “disinformation” because we argue that science rejecters, for the most part, are convinced of the validity of the (incorrect) information they share publicly, aiming to counteract the dissemination of information by scientists, but do not intend to deliberately mislead users (or, at least, we cannot be sure about any intentions to purposely cause harm) [11,12].

Science rejection develops particularly in those areas where science has led to discoveries that threaten individual lifestyles or worldviews or affect the interests of companies [3]. Particularly high religiosity, conservative political attitudes, and low science literacy have been identified as predictors of science skepticism in previous studies [6]. However, the use of social media may also play a role in developing tendencies for science rejection. Recent research suggests that social media has become one of the most widely used sources of health-related information, particularly among young people [13,14]. This is because social media bypasses traditional “gatekeeping” roles typically filled by professional journalists and editors [15] and may be a particularly interesting source for those who already mistrust established media organizations and governments [8]. Without these checks, unverified or misleading information can circulate widely, potentially eroding public trust in scientific consensus and established knowledge [4].

Several content analyses have shown that for some specific scientific topics, particularly those related to public health, such as COVID-19 or vaccinations, a considerable amount of information found on social media is incorrect or inaccurate [16-18]. Accordingly, social media use was found to be associated with belief in COVID-19 misinformation [19], hesitancy to vaccinate against COVID-19 [20], and low trust in government [21] and other institutions [8]. Furthermore, the use of YouTube (Google) in particular was found to be associated with distrust in science and research [4]. Concerns have been raised that the combination of inconsistent quality of online health information and distrust of evidence-based sources of information may lead to negative public health outcomes, such as delays in seeking essential medical care or pursuing potentially harmful alternative therapies [22].

One of the primary sources of information social media users are confronted with on these platforms are the so-called social media influencers [5,8,23]. They are considered role models, particularly among young people and those with high mistrust in established media organizations and the government [8,24]. While there is some evidence from cross-sectional studies showing that distrust in science and research is associated with the use of YouTube [4], and experimental designs that assessed the effects of specific misinformation topics [5], there are, so far, no experimental studies available that explored the impact of a video by a social media influencer rejecting core principles of science on users’ attitudes toward science or trust in scientists.

Social media influencers are typically considered for their persuasive potential in marketing efforts [25]. Recently, however, there has been a growing interest in influencers’ content on political and social issues [26]. People are increasingly turning to social media for information, especially those who are skeptical of mainstream media [8]. Social media influencers, in particular, can be seen as modern-day opinion leaders. In the traditional understanding of opinion leadership, opinion leaders are those who are better informed about certain issues and therefore give advice on these issues to others [27]. Social media influencers can now act as parasocial (as there is no actual face-to-face contact between influencers and their followers) opinion leaders for their audiences [28]. Thus, they use their platform to share their opinions and views, often in a very relatable and approachable way, which makes the audience more likely to adopt the shared views [29]. Based on the role these social media influencers may take in their audience’s perception, it is plausible to assume that exposure to a video by a social media influencer rejecting core principles of science might have negative effects on users’ attitudes toward science or trust in scientists.

### Strategies for Effective Responses to Science Rejection

The prevalence of inaccurate information on science and scientific research on social media and the potential detrimental impact of such portrayals on society raises the question of how to react to videos that feature false or negative portrayals of science or discredit and reject science on social media platforms (eg, YouTube). Besides top-down approaches such as technical or legal countermeasures (ie, content moderation by platform providers, deplatforming of influencers, or integration of content disclosures based on keywords), there are bottom-up approaches that rely on the intervention of other users in the comments section, also known as bystander intervention [30]. The general rule of thumb in research on crisis communication is that any response to criticism or negative publicity is better than no response at all [10,31], but, so far, there is only limited evidence of how user comments should look like in order to effectively counter postings with false claims about science on social media.

One source of information that may inform strategies on how to effectively react to science rejection stems from literature on debunking myths and countering misinformation related to specific topics of scientific research. Most of these studies are related to myths and misinformation about COVID-19. A meta-analysis that explored factors underlying effective messages to counter attitudes and beliefs based on misinformation revealed that the effectiveness of messages was mainly determined by the number of details included in the message. Debunking was more successful when the counter message contained elaborate information on the respective issue as opposed to just labeling misinformation as incorrect [32]. It is, however, important not to repeat the respective myth when informing audiences about misinformation but to emphasize the correct information [33]. Combining information and social norm modeling with myth-busting has been found to be effective in combating misinformation and distrust related to COVID-19 vaccines [34]. Similarly, briefly viewing an infographic about science (ie, pictures that highlighted that scientific recommendations change along with newly available evidence) increased trust in science, which in turn reduced incorrect beliefs in COVID-19 misinformation [35]. In a study on the effectiveness of countering conspiracy theories related to COVID-19 [36], a video featuring a lecture on nature and features of conspiracy theories, as well as the debunking of some widely spread conspiracy theories, reduced political radicalization. In contrast, highlighting the lack of credibility of the source of the conspiracy theory prevented the intensification of radicalization but did not reduce previous radicalization [36].

Several authors have put together lists of recommendations for communication practitioners and members of the scientific community on how to approach public debates with individuals who are skeptical of research findings or respond to misinformation based on current literature about debunking misinformation [3,15,37]. However, there is little evidence on how to react to individuals who are not just skeptical toward specific research findings or provide misinformation on particular topics, but completely deny or reject core principles of science. Schmid and Betsch [10] differentiated two general types or goals of rebuttal messages to science rejection: (1) responding to misinformation by supporting the scientific standpoint with scientific facts (ie, topic rebuttal) and (2) refuting the opposing position by attacking its plausibility and unmasking its illegitimate techniques (ie, technique rebuttal). The authors found that both approaches had positive effects on audiences’ beliefs in scientific evidence, but neither one of the two approaches nor a specific combination of them was overall superior in terms of effectiveness [10]. However, more research on the effectiveness of specific strategies or approaches to respond adequately to science rejection is needed.

Furthermore, all available studies that may inform effective responses to science rejection have focused on one of the following two approaches: (1) they have either explored what scientists, researchers, or practitioners can do to ideally state their case or persuade the audience in a public debate [3,10,15,37] or (2) they have exposed participants in experimental settings to news articles, videos, or infographics that addressed well-known or preinduced myths or misinformation [32-36]. However, as mentioned above, the majority of incorrect information about scientific topics, or materials that support science rejection, is not transported in traditional media or public debates, but on social media platforms such as YouTube [4,15]. Social media platforms usually have a commenting system allowing users to share comments. These comments represent opinions, queries, appreciations, or displeasure regarding the provided or shared content [38]. Critical user comments regarding content that rejects science may play an important role in mitigating the detrimental effects of such postings, but there are no studies available testing whether the different types of user comments are effective in countering content of social media influencers who openly reject science.

In this study, we conducted a randomized controlled online trial in order to pursue the following two objectives: (1) to explore the effects of an online video on social media where an influencer openly rejects facts of (medical) science on users’ attitudes toward science and scientists and (2) to assess to what extent different response strategies by other users in the commentary section change these effects.

Based on the aforementioned concept of opinion leadership [27], it appears plausible to assume that users’ attitudes toward science and scientists might be influenced by a video of a social media influencer openly rejecting science as compared to a video that is unrelated to science. Furthermore, according to the negativity bias, news with negative overtones, such as criticism or accusations from social media users that challenge fundamental components of modern society (eg, science and research), become more salient, are subsequently processed with greater attention, and therefore have a stronger impact on audiences’ arousal, perceptions, attention, and learning than positive contents [39,40]. Based on these theoretical concepts, we assumed that users’ attitudes toward science and scientists will be impacted by a science rejection video as compared to a video unrelated to science. However, since evidence on the impact of misinformation on social media is mixed [5] and there are no previous studies that specifically addressed this particular research question, we assumed that any direction of the impact of the science rejection video might be possible in principle. For example, it is possible that the influencer’s rejection of science would legitimize skepticism toward scientific research and thereby negatively affect attitudes toward science and scientists and decrease support, trust, and interest related to scientific research. At the same time, however, it may also be the case that openly rejecting science in its entirety is viewed as controversial by audiences, triggering the opposite effect, especially among more educated viewers who value science. If this is the case, the messages might increase support for scientists and interest in scientific research. In fact, such a message might evoke psychological reactance among audiences, which is defined as a motivational state to regain personal freedom when individuals experience a threat to their freedom [41]. Dogmatic or extreme messages that challenge personal belief systems can function as such a threat, potentially resulting in a boomerang effect [42]. Thus, since both positive and negative effects are theoretically plausible for most outcomes, we formulated the following open, nondirectional hypothesis:

- Hypothesis 1: The outcomes (1.1) trust in scientists, (1.2) attitudes toward scientific research, (1.3) science support, (1.4) interest in scientific research, (1.5) belief in science, (1.6) conspiracy beliefs, and (1.7) evaluation of video and influencer will differ between the groups based on the differences in the content of the videos (ie, science rejection content compared to no science content).

The majority of available literature on countering hate speech, debunking of misinformation, and mitigating reputational damage seems to suggest that educating the public about an issue by focusing on providing factual information is the most effective response to science rejection [30,32,34,36,43]. This is consistent with the misinformation receptivity framework, which highlights the importance of perceived credibility of the source and perceived plausibility of information as characteristics for messages effectively refuting misinformation [44,45]. Based on this model, we thus propose that factual counterarguments that plausibly refute the arguments presented in a video could be a relevant strategy for addressing science rejection. In addition, rejecting the credibility of the source through personal attacks, such as describing the source as unintelligent or misinformed, could dampen the persuasiveness of what that person has shared. Also, a combination of both strategies should help to refute science rejection content. However, evidence on the effectiveness of refutations is mixed [45], and there is no “golden standard” or clear guidelines in the development of rebuttals to science rejection [10]. Thus, we formulated to following nondirectional hypothesis:

- Hypothesis 2: The outcomes (2.1) trust in scientists, (2.2) attitudes toward scientific research, (2.3) science support, (2.4) interest in scientific research, (2.5) belief in science, (2.6) conspiracy beliefs, and (2.7) evaluation of video, influencer, and comments will differ between the groups based on the differences in the user comments provided as response to the videos (ie, personal attacks on the influencer vs responses providing primarily factual information vs a mix of both strategies vs no response).

## Methods

### Participants

Between May 21, 2024, and June 27, 2024, we conducted a double-blind randomized controlled trial that followed strict intent-to-treat principles [46]. Participants were recruited via emails with invitations to participate in an online study on the impact of videos by influencers on social media sent to registered adult members of the German noncommercial online access panel SoSci Survey. The SoSci Survey panel comprises approximately 100,000 active individuals from German-speaking countries who registered for the panel pool via a double opt-in procedure [47]. The invitation emails were sent to 4000 panelists by the administrators of SoSci Survey and included basic information on the topic and (expected) duration of the study, but did not reveal any details. Researchers and participants remained blinded to the assignment of the experimental conditions until the end of data collection.

Of the 470 participants who responded to items on sociodemographics, 261 (55.5%) identified themselves as women and 199 (42.3%) as men, 7 (1.5%) indicated other genders, and 3 (0.6%) opted not to reveal their gender. The participants’ ages ranged from 18 to 88 years, with a mean age of 49.1 (SD 15.8) years. In terms of the highest completed education for these 470 participants, 92 (19.6%) indicated educational attainment below high school graduation, 136 (28.9%) indicated they had a high school diploma, and 242 (51.5%) had completed college or university. Table 1 lists an overview of participants’ sociodemographic characteristics in each experimental condition. There were no differences between the 5 experimental conditions in terms of sociodemographics.

**Table 1:** Overview of descriptive statistics of sociodemographic variables for all experimental conditions in the online randomized controlled trial (N=470).

Variables				Control (n=94)		No response (n=92)		Attack response (n=94)		Factual response (n=93)			Mixed response (n=97)			*P* value
Age (years), mean (SD)				51.1 (15)		49.9 (15)		47.9 (17)		47.3 (17.3)			49.4 (14.8)			.48^a^
**Sex, n (%)**																
	Female		48 (51.1)		53 (57.6)		48 (51.1)		53 (57)		59 (60.8)			.58^b^		
	Male		43 (45.7)		38 (41.3)		45 (47.9)		38 (40.9)		35 (36.1)			.51^b^		
	Other		3 (3.2)		1 (1.1)		0 (0)		2 (2.2)		1 (1)			.45^b^		
	Undisclosed		0 (0)		0 (0)		1 (1.1)		0 (0)		2 (2.1)			.52^b^		
**Education, n (%)**																
		College or university	54 (57.4)		56 (60.9)		43 (45.7)		44 (47.3)			45 (46.4)			.11^b^	
		High school	25 (26.6)		19 (20.7)		34 (36.2)		27 (29)			31 (32)			.19^b^	
		Below high school	15 (16)		17 (18.5)		17 (18.1)		22 (23.7)			21 (21.6)			.70 ^b^	

^a^*P* values calculated by ANOVA assessing group differences (*F*_4,465_=0.88).

^b^*P* values calculated by Fisher exact test assessing group differences.

### Power Analysis

According to a sample size calculation with G*Power (version 3.1.9.7; Franz Faul, Universität Kiel) [48], an ANOVA in a design with 5 experimental conditions along with an error probability of 0.05 and a power of 0.80 will require a minimum of 395 participants in order to detect a small to medium treatment effect of *f*=0.175, which is slightly more conservative, but comparable, to common benchmarks recommended and used for exploratory psychological research [49-53].

### Materials

Participants of the intervention groups watched selected footage (length 4 minutes and 9 seconds) of a video entitled “Why science is nothing but a lie – you will change your way of thinking” of a German YouTube influencer [54]. At the beginning of the footage presented to the participants, the influencer claims that scientific research is based mostly on “false facts” and lies, and uses medical science, infectious diseases in particular, as an example for his claims. He explains that conventional medicine is based on the work of Robert Koch and Louis Pasteur, who proposed far-fetched ideas, which were never proven to be true or accurate, but were considered as facts by authorities for the purpose of imposing fear in the population in order to make money and maintain power. The influencer then continues to claim there is no progress in science, because its foundation is based on lies, and that most people, including scientists, do not care about this issue or are too narrow-minded to notice it. The footage concludes with the influencer claiming that everyone who dares to speak up is getting shut off or suppressed by authorities. At no point in this video does the influencer present any evidence that would support his claims.

After viewing the footage, the participants were directed to a webpage that provided comments on the video by other YouTube users. All comments were fictitious but loosely based on real comments on the actual video, and contained between 45 and 376 characters (including emoticons). Based on response strategies to science denialism in public discussions tested in a previous study [10], factors stated in the misinformation receptivity framework [44], highlighting the importance of perceived credibility of the source and perceived plausibility of information, and observations of different types of actual responses to the science rejection video on YouTube, we developed 4 different types of responses to the video in this study: responses providing primarily personal attacks on the influencer, responses providing primarily factual information countering the content in the video, a mix of both strategies, and no response. Since the reactions to these types of videos by users on YouTube are usually very diverse (ie, there is never a unanimous approval or rejection of such videos among users), we provided a balanced mixture of supporting and opposing comments to the participants in the intervention groups (except for the “No response” group).

Intervention group 1 (Attack response) read 8 comments posted as a response to the video. In total, 4 of them were supportive and contained statements such as “What a great, honest talk!” “I confirm this as an awakened doctor!” and “Thank you for your valuable work!” In addition, 4 comments criticized the content of the video and attacked the influencer on a personal level. These comments contained statements such as “You have no idea what you’re talking about, but you feel great because you finally have an excuse for your lack of education,” “On what insights do you practice medicine? Probably only on your own experiences!” or “My morals won't allow a small sports influencer who thinks he's got it all figured out to spread his dose of misinformation to the ignorant.”

Intervention group 2 (Factual response) read the same 4 supportive comments as Intervention group 1, but 4 different opposing comments. These comments focused on criticizing and refuting the video content by providing primarily factual information. They contained statements such as “Thanks to Robert Koch, we know the causes of tuberculosis and cholera and can treat them,” “In science, it is always made transparent which methodology was used, what was investigated, etc,” or “Without the progress that humanity has made thanks to science, we would...still die early from infections of small wounds.”

Intervention group 3 (Mixed response) read the same 4 supportive comments and a mix of opposing comments (ie, 2 comments that attacked the influencer and 2 comments with factual information) from intervention groups 1 and 2.

Intervention group 4 (No response) read a statement informing the participants that this video did not have any user comments. Participants of the control group watched a video (length 4 minutes and 6 seconds) with a similar style (ie, both videos feature a young male influencer lecturing his audience about a specific topic by talking directly into the camera) and length (ie, both videos had a length of approximately 4 minutes) as the intervention video, but unrelated to science and research. The video contained footage of an influencer discussing how to properly shine shoes [55].

### Procedure

Once informed consent was obtained on the first page of the online survey, we collected participants’ sociodemographic data. Afterwards, all participants were randomly assigned to 1 of the 5 experimental conditions via automated urn randomization with an even allocation ratio as outlined by Till et al [56]. After watching the respective footage and reading the respective response, we used questionnaires to collect data on all outcomes (ie, trust in scientists, attitudes toward scientific research, science support, interest in scientific research, belief in science, conspiracy beliefs, and evaluation of video, influencer, and user comments). Subsequently, all participants completed 1 item for the manipulation check and blinding success, respectively. The online survey concluded with an extensive debriefing and clarification of the misinformation provided in the intervention video.

### Measures

#### Trust in Scientists

We used 5 items taken from the Special Eurobarometer 516 survey [57] to assess trust in scientists. Since these items do not comprise a validated scale, we used Cronbach α to assess the internal consistency of the items and excluded items with low coefficients. As a result, we assessed trust in scientists with the following 3 items: “We can no longer trust scientists to tell the truth about controversial scientific and technological issues because they depend more and more on money from industry,” “Scientists only look at very specific issues and do not consider problems from a wider perspective,” and “Nowadays, the problems we are facing are so complex that scientists are no longer able to understand them.” These items were rated on a scale from 1 (strongly disagree) to 5 (strongly agree). We calculated mean scores across all 3 items and reversed the coding in order to have higher scores indicating greater trust in scientists (mean 3.76, SD 0.87; score range 1-5; Cronbach α=0.75).

#### Attitudes Toward Scientific Research

We used 9 items taken from the Special Eurobarometer 516 survey [57] to assess respondents’ attitudes toward scientific research. Exclusion of 2 items due to low internal consistency resulted in 7 items used for the final statistical analysis (eg, “Science can sort out any problem” or “Young people’s interest in science is essential for our future prosperity”), which were rated on a scale from 1 (strongly disagree) to 5 (strongly agree). We calculated mean scores across all 7 items, with higher scores indicating greater approval of science (mean 3.33, SD 0.53; score range 1-5; Cronbach α=0.53). Note that, because of the scale’s low reliability, which remained low even after omitting individual items with low coefficients, we excluded this measure from the statistical data analysis.

#### Science Support

Participants’ science support was assessed with the following item, which was loosely based on a measure developed by Rutjens et al [6]: “In Germany, Austria, and Switzerland, an average of approximately 3% of the respective gross domestic product (GDP) is spent on research. Please indicate to what extent you believe the government should spend more or less than 3% of GDP on scientific research in the future.” Participants provided their ratings on a visual analog scale with a 2-sided scroll bar ranging from 0 (much less) to 100 (much more). Higher scores indicate greater science support (mean 76.34, SD 17.03; score range 0-100).

#### Interest in Scientific Research

Participants’ interest in scientific research was assessed with the following single item: “In everyday life, we have to deal with many different problems that we are more or less interested in. Please indicate to what extent you are interested in science.” Participants provided their ratings on a visual analog scale with a 2-sided scroll bar ranging from 0 (not at all interested) to 100 (very interested). Higher scores indicate greater interest in scientific research (mean 80.43, SD 17.53; score range 0-100).

#### Belief in Science

We used a singular item taken from the Special Eurobarometer 516 survey [57] to assess respondents’ belief in science: “How do you think is the overall influence of science and technology on society?” which was rated on a scale from 1 (very negative) to 4 (very positive). Higher scores indicate greater belief in science (mean 3.19, SD 0.53; score range 1-4).

#### Conspiracy Beliefs

We used a scale developed by Nocun and Lamberty [58] consisting of 5 items (eg, “There are secret organizations that have a major influence on political decisions”) to assess conspiracy beliefs. These items were rated on a scale from 1 (strongly disagree) to 7 (strongly agree). We calculated mean scores across all 5 items, with low scores indicating a lack of critical world view, average scores indicating a healthy amount of criticism, and higher scores indicating greater conspiracy beliefs (mean 2.86, SD 1.33; score range 1-7; α=0.80).

#### Evaluation of Video and Influencer

We asked participants to assess the video they had watched as well as the influencer with the following 4 single items: “How much did you like the video?” “How informative was the video?” “How much did you like the influencer?” and “How trustworthy was the influencer?” All items were rated on a scale from 1 (not at all) to 5 (very much). Higher scores indicated greater approval of the video or influencer (item 1: mean 1.78, SD 1.10; item 2: mean 1.84, SD 1.21; item 3: mean 2.30, SD 1.24; and item 4: mean 1.97, SD 1.32; score range 1-5).

#### Evaluation of Comments

We asked participants of intervention groups 1-3 (the “No response” group and the control group were not exposed to any comments) to assess the comments that criticized the video with the following three single items: “How much did you like the comments?” “How informative were the comments?” and “How trustworthy were the comments?” All items were rated on a scale from 1 (not at all) to 5 (very much). Higher scores indicated greater approval of the comments (item 1: mean 3.13, SD 1.15; item 2: mean 2.68, SD 1.21; and item 3: mean 3.12, SD 1.26; score range 1-5).

#### Masking

In order to mask the aim of this study and the group assignment, we included 8 items that asked participants to rate the importance of science as well as fashion for their personal life (eg, “I often talk about science/fashion with other people”). All items were rated on a scale from 1 (strongly disagree) to 5 (strongly agree).

#### Blinding Success

In order to assess the success of blinding manipulation, we asked participants to indicate which group they thought they had been allocated to (intervention group, control group, or don’t know) as outlined by Kolahi et al [59].

#### Manipulation Check

In order to assess whether the experimental manipulation had been successful, we asked participants to indicate how personally offensive or factual the comments on the video were on a visual analog scale with a 2-sided scroll bar ranging from 0 (personally offensive) to 100 (factual), with higher scores indicating greater perception of the comments being factual (mean 46.09, SD 25.57; score range 0-100).

### Data Analysis

For the primary data analysis, the effects of the intervention on all outcomes were compared between the 5 experimental conditions (ie, Attack response, Factual response, Mixed response, No response, and control group) with univariate ANOVAs along with Bonferroni-corrected contrast tests for the examination of individual group differences, testing both hypothesis 1 and hypothesis 2. In an additional exploratory analysis that specifically focused on the impact of the science rejection video compared to the control group, we compared outcomes of all intervention groups combined with the control group in order to explore the impact of the science rejection video compared to the control group with unpaired 2-sample *t* tests. Differences in sociodemographics between the experimental conditions as well as between survey completers and dropouts were analyzed with Fisher exact tests, unpaired 2-sample *t* tests, and univariate ANOVAs.

### Ethical Considerations

This study was reviewed and approved by the Research Ethics Board of the Medical University of Vienna (study protocol 1206/2024). All statistical analyses were conducted within the scope of this ethics approval. All participants provided informed consent electronically before the start of the study. No personally identifiable information was collected, and no compensation was offered for participation. The study was preregistered with the German Clinical Trial Registry as DRKS00033829.

## Results

### Overview

**Figure 1:**
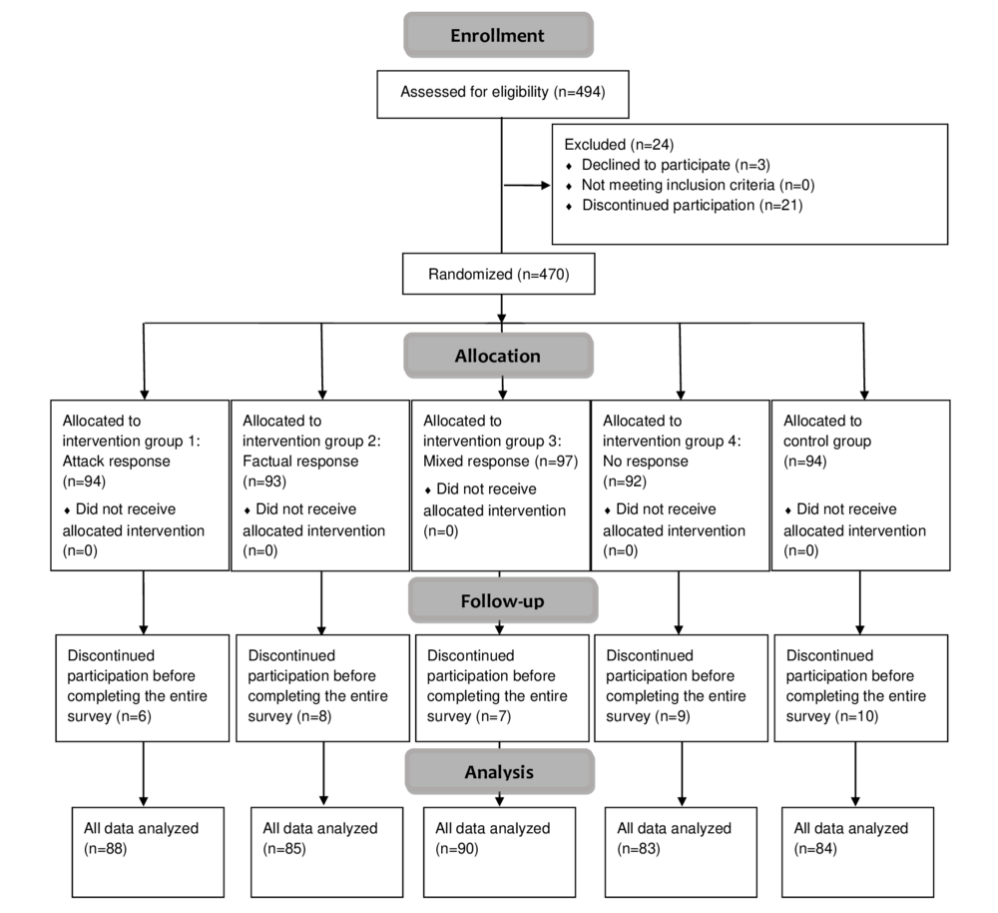
CONSORT study flowchart showing participant allocation, interventions, and analysis.

Figure 1 demonstrates an illustration of the study flowchart as per the CONSORT (Consolidated Standards of Reporting Trials) guidelines (CONSORT checklist in Multimedia Appendix 1). Of the 494 individuals who accessed the survey, 3 (0.6%) individuals did not provide informed consent, and 21 (4.3%) discontinued their participation before randomization was completed. The remaining 470 (95.1%) individuals were randomly allocated to 1 of the 5 experimental conditions (“Attack response” group: n=94; “Factual response” group: n=93; “Mixed response” group: n=97; “No response” group: n=92; and control group: n=94) and included in the statistical analysis of the study. A total of 430 of 470 (91.5%) randomized participants completed the entire survey.

Only 3 respondents were already familiar with the influencer in the science rejection video before the current intervention (one in the “Attack response,” “Mixed response,” and “No response” group, respectively), and none of the respondents in the control group were familiar with the influencer in the control video. Furthermore, 1 respondent (in the “Attack response” group) has already seen footage from the science rejection video before the study. The overwhelming majority of the participants thus did not have a preexisting relationship with the influencer.

### Comparisons With Dropouts

There were no differences between participants who completed the entire study and those who were randomized, but dropped out of the study before completion, with regard to gender (*P*=.26), age (t_468_=–0.67; *P*=.50), education (*P*=.17), and group allocation (*P*=.83). Similarly, there were no differences between randomized participants and dropouts before randomization in terms of gender (*P*=.88), age (t_487_=–0.49; *P*=.62), and education (*P*=.17).

### Blinding Success

A total of 430 participants responded to the item assessing blinding success. Out of these 430 participants, 117 (27.2%) correctly guessed their group allocation, whereas 94 (21.9%) provided an incorrect answer, and 219 participants (50.9%) responded with “don’t know.” With the number of “don’t know” responses being relatively high as well as correct and incorrect guesses being balanced, blinding was considered successful [57].

### Manipulation Check

There was a difference in the perception of the provided user comments being factual or personally offensive between intervention groups 1-3 (*F*_2,261_=7.14, η_p_^2^=.05, *P*<.001). Participants of the “Factual response” group (mean 48.53, SD 22.68) perceived the comments as significantly more factual than participants of the “Attack response” group (mean 34.89, SD 22.18; Cohen *d*=0.23, Cohen *d* score range 0.08-0.38; *P*<.001). The score of the “Mixed response” group (mean 43.07, SD 26.10) was lower than in the “Factual response” group (Cohen *d*=–0.11, Cohen *d* score range –0.03 to 0.04; *P*=.22), and higher than in the “Attack response” group (Cohen *d*=0.12, Cohen *d* score range –0.02 to 0.27; *P*=.13), although these differences did not reach statistical significance. Based on these scores, the experimental manipulation was considered successful. There are no results regarding the “No response” group and the control group, as these groups did not read any comments.

### Primary Analysis: Differences Between Experimental Conditions

We tested both hypothesis 1 and hypothesis 2 by comparing all outcomes between the 5 experimental conditions. Table 2 shows an overview of comparisons of trust in scientists, science support, interest in scientific research, belief in science, conspiracy beliefs, and evaluation of video and influencer between the 5 experimental conditions. An overview of differences in the evaluation of the user comments between groups 1-3 (ie, “Attack response” group, “Factual response” group, and “Mixed response” group) is provided in Table 3.

The experimental conditions had no significant effect on trust in scientists (*F*_4,427_=1.33, η_p_^2^=.01; *P*=.26), science support (*F*_4,426_=0.15, η_p_^2^=.001; *P*=.96), interest in scientific research (*F*_4,426_=1.78, η_p_^2^=.02; *P*=.13), belief in science (*F*_4,426_=0.71, η_p_^2^=.007; *P*=.58), and conspiracy beliefs (*F*_4,426_=0.30, η_p_^2^=.003; *P*=.88). Based on these results, we rejected hypothesis 1.1 to hypothesis 1.6 as well as hypothesis 2.1 to hypothesis 2.6.

**Table 2:** Overview of means of trust in scientists, science support, interest in scientific research, belief in science, conspiracy beliefs, and evaluation of video and influencer across all experimental conditions, as well as overview of the mean differences of all individual intervention groups with the control group in the online randomized controlled trial.

Experimental conditions		Control	No response			Attack response			Factual response			Mixed response	
		Mean score (95% CI)	Mean score (95% CI)	Mean difference (95% CI)^a^	Mean score (95% CI)		Mean difference (95% CI)^a^	Mean score (95% CI)		Mean difference (95% CI)^a^	Mean score (95% CI)^a^		Mean difference (95% CI)^a^
**Outcomes**													
	Trust in scientists (Cronbach α= 0.75; n=432)	3.57 (3.26 to 3.77)	3.78 (3.59 to 3.97)	0.21 (–0.17 to 0.59)	3.78 (3.60 to 3.95)		0.21 (–0.17 to 0.58)	3.85 (3.68 to 4.01)		0.28 (–0.10 to 0.65)	3.81 (3.62 to 4.01)		0.25 (–0.13 to 0.62)
	Science support (n=431)	76.96 (72.93 to 81.00)	75.66 (71.62 to 79.71)	–1.30 (–8.77 to 6.17)	77.18 (74.05 to 80.31)		0.22 (–7.14 to 7.58)	76.21 (72.78 to 79.64)		–0.75 (–8.18 to 6.67)	75.70 (72.06 to 79.34)		–1.26 (–8.56 to 6.04)
	Interest in scientific research (n=431)	84.00 (80.56 to 87.44)	82.30 (79.12 to 85.48)	–1.70 (–9.33 to 5.93)	79.05 (75.10 to 82.99)		–4.96 (–12.47 to 2.56)	78.59 (74.65 to 82.53)		–5.41 (–12.99 to 2.17)	78.47 (74.45 to 82.49)		–5.53 (–12.99 to 1.93)
	Belief in science (n=431)	3.14 (3.03 to 3.26)	3.24 (3.14 to 3.35)	0.10 (–0.13 to 0.33)	3.17 (3.06 to 3.28)		0.03 (–0.20 to 0.26)	3.24 (3.12 to 3.35)		0.09 (–0.14 to 0.32)	3.14 (3.02 to 3.26)		0.00 (–0.23 to 0.23)
	Conspiracy beliefs (Cronbach α=0.80; n=431)	2.98 (2.72 to 3.24)	2.90 (2.59 to 3.21)	–0.08 (–0.66 to 0.51)	2.84 (2.59 to 3.10)		–0.13 (–0.71 to 0.44)	2.76 (2.49 to 3.04)		–0.21 (–0.79 to 0.37)	2.83 (2.52 to 3.15)		–0.14 (–0.71 to 0.43)
**Evaluation of video and influencer (n=432)**													
	How much did you like the video?	3.02 (2.78 to 3.27)	1.61 (1.41 to 1.82)	–1.41 (–1.81 to –1.01)^b^	1.47 (1.31 to 1.62)		–1.56 (–1.95 to –1.17)^b^	1.42 (1.23 to 1.61)		–1.61 (–2.00 to –1.21)^b^	1.42 (1.24 to 1.59)		–1.61 (–2.00 to –1.22)^b^
	How informative was the video?	3.49 (3.26 to 3.72)	1.54 (1.31 to 1.78)	–1.95 (–2.34 to –1.56)^b^	1.40 (1.25 to 1.55)		–2.09 (–2.48 to –1.71)^b^	1.51 (1.33 to 1.69)		–1.98 (–2.36 to –1.59)^b^	1.35 (1.19 to 1.51)		–2.14 (–2.52 to –1.75)^b^
	How much did you like the influencer?	3.69 (3.46 to 3.92)	2.14 (1.90 to 2.39)	–1.55 (–2.00 to –1.09)^b^	1.98 (1.77 to 2.19)		–1.71 (–2.16 to –1.27)^b^	1.97 (1.74 to 2.19)		–1.73 (–2.17 to –1.28)^b^	1.79 (1.59 to 2.00)		–1.90 (–2.34 to –1.46)^b^
	How trustworthy was the influencer?	3.98 (3.76 to 4.19)	1.64 (1.44 to 1.84)	–2.34 (–2.72 to –1.96)^b^	1.45 (1.29 to 1.61)		–2.52 (–2.90 to –2.15)^b^	1.47 (1.27 to 1.66)		–2.51 (–2.89 to –2.13)^b^	1.37 (1.22 to 1.53)		–2.60 (–2.98 to –2.23)^b^

^a^Comparison of means with the control group with Bonferroni corrected contrast tests.

^b^Mean differences with significant *P* values <.05.

**Table 3:** Overview of means and mean differences in evaluation of the comments between intervention groups 1-3 in the online randomized controlled trial (RCT).

Comments^a^	Attack response	Factual response			Mixed response		
	Mean score (95% CI)	Mean score (95% CI)	Mean difference (95% CI)^b^	Mean score (95% CI)		Mean difference (95% CI)^b^	Mean difference (95% CI)^c^
How much did you like the comments? (n=265)	2.90 (2.65 to 3.15)	3.21 (2.98 to 3.44)	0.31 (–0.11 to 0.73)	3.27 (3.03 to 3.52)		0.38 (–0.03 to 0.79)	0.07 (–0.35 to 0.48)
How informative were the comments? (n=265)	2.40 (2.16 to 2.64)	2.85 (2.59 to 3.10)	0.45 (0.01 to 0.89)^d^	2.80 (2.54 to 3.06)		0.40 (–0.03 to 0.84)	–0.05 (–0.48 to 0.39)
How trustworthy were the comments? (n=265)	2.78 (2.51 to 3.06)	3.28 (3.02 to 3.54)	0.50 (0.04 to 0.95)^d^	3.30 (3.04 to 3.55)		0.51 (0.07 to 0.96)^d^	0.02 (–0.43 to 0.47)

^a^There are no results regarding the “No response” group and control group, as these groups did not read any comments.

^b^Comparisons of means with the “Attack response” group with Bonferroni corrected contrast tests.

^c^Comparisons of means with the “Factual response” group with Bonferroni corrected contrast tests.

^d^Mean differences with significant *P* values <.05.

In contrast, the experimental conditions had a significant effect on how much the participants liked the video (*F*_4,427_=49.40, η_p_^2^=.32; *P*<.001), how much they found the video to be informative (*F*_4,427_=88.32, η_p_^2^=.45; *P*<.001), how much they liked the influencer (*F*_4,427_=48.19, η_p_^2^=.31; *P*<.001), and how much they found the influencer to be trustworthy (*F*_4,427_=138.43, η_p_^2^=.57; *P*<.001). Bonferroni corrected contrast tests indicated that scores for liking the video (Cohen *d*=0.46-0.54) finding the video informative (Cohen *d*=0.65-0.72), liking the influencer (Cohen *d*=0.45-0.56), and finding the influencer trustworthy (Cohen *d*=0.79-0.90) were higher in the control group than in all intervention groups, whereas there were no differences between the individual intervention groups (ie, groups 1-4). These results supported hypothesis 1.7, as video and influencer were rated more favorably across all domains in the control group than in all intervention groups.

Furthermore, in terms of assessment of the comments, there were differences between intervention groups 1-3 in participants’ assessment of how informative (*F*_2,262_=3.78, η_p_^2^=.03; *P*=.02) and how trustworthy (*F*_2,262_=4.86, η_p_^2^=.04; *P*=.01) the comments were. Bonferroni corrected contrast tests indicated that participants of the “Factual response” group rated the comments as more informative than in the “Attack response” group (Cohen *d*=0.15, Cohen *d* score range 0.004-0.30; *P*=.04). Furthermore, participants of the “Factual response” group (Cohen *d*=0.16, Cohen *d* score range 0.01-0.31; *P*=.03) and the “Mixed response” group (Cohen *d*=0.17, Cohen *d* score range 0.02-0.32; *P*=.02) rated the comments as more trustworthy than those in the “Attack response” group. There were no group differences in terms of liking the comments (*F*_2,262_=2.77, η_p_^2^=.02; *P*=.07). These results partly supported hypothesis 2.7, as we found some differences in the evaluation of the user comments between individual intervention groups.

### Exploratory Analysis: The Impact of the Science Rejection Video Compared to the Control Group

Table S1 in Multimedia Appendix 2 lists the randomization checks, specifically comparing the pooled treatment groups versus the control group. There were no differences between these two conditions in terms of sociodemographics. Table S2 in Multimedia Appendix 2 provides an overview of binary comparisons of participants’ outcomes in all groups who watched the science rejection video (ie, groups 1-4 combined) compared to the control group. Trust in scientists was overall higher in the intervention groups than in the control group, whereas interest in scientific research was lower in the intervention groups than in the control group. There were, however, no differences in terms of science support, belief in science, and conspiracy beliefs. Finally, consistent with the results of the primary analysis, assessment of the video and the influencer was less favorable in the intervention groups than in the control group for all items.

## Discussion

### Principal Findings

This study assessed the impact of a YouTube video by a social media influencer who openly rejects science on users’ attitudes toward science and scientists, and to what extent different response strategies applied in user comments changed these effects. When we compared all 5 experimental conditions with each other in the primary analysis, we found that participants rated the video and the influencer less favorably in the science rejection conditions than in the control group. However, the analysis revealed no differences between the experimental conditions in terms of all other outcomes, suggesting that participants of this study were largely not negatively impacted by the science rejection video. This offers a nuanced view of opinion leadership of influencers and aligns with a recent experimental study on COVID-19–related misinformation by an influencer, which found that such misinformation did not consistently lead to misunderstandings about SARS-CoV-2 among audiences [5].

Furthermore, the different responses to the science rejection video (ie, factual, attack, or mixed) did not seem to impact participants’ trust in scientists, science support, interest in research, belief in science, conspiracy beliefs, or evaluations of the video and the influencer. A potential explanation for the lack of differing effects of the experimental conditions on the respondents might be that the views expressed in the science rejection video were so extreme that the specific message of the influencer was considered entirely irrelevant to their own views. Similarly, in the light of this strong divergence from their own opinions, it might have made little difference what others wrote about the video content in the comments, as once existing, strongly entrenched beliefs can also prevail even if confronted with nonsupportive views [60]. Consistent with cognitive dissonance theory [61], it might also be the case that the content of the science rejection video was perceived as attitude-challenging information by the majority of participants in this sample with relatively high education levels, inducing dissonance and thus limiting the impact of the video [62]. Furthermore, a single exposure to a short video might not have been sufficient to change opinions and attitudes toward fundamental beliefs such as the belief in the validity of science. It has been noted in the literature that individual core beliefs, particularly those related to politics, health, and social issues, are often too robust to change after a brief media intervention [63,64].

Despite this, factual responses tended to be perceived as more informative and trustworthy than personal attacks on the influencer in this study, suggesting that supporting the scientific standpoint with factual information [10] that refutes the plausibility of the information the influencer has shared [44] may have been perceived more favorably by the respondents. This aligns with research emphasizing the importance of educating the public with factual responses about science rejection [30,32,34,36,43]. While none of the response strategies significantly altered how the video was perceived by participants in this study, previous work advocates for constructive, transparent, and persistent engagement in public debates on science [3,37].

When we compared the outcomes of all intervention groups combined with the control group in an additional exploratory analysis, we found that, contrary to our expectations, participants who were exposed to the science critical video reported greater trust in scientists than participants of the control group. In accordance with the principle of cognitive consistency, individuals are generally motivated to maintain coherence in their beliefs and attitudes within a stable psychological framework [61]. It is therefore possible that exposure to content rejecting science may have resulted in a boomerang effect [65] in the current sample, which consisted mainly of individuals with a high affinity for scientific research (ie, high education and high interest in scientific research), and increased participants’ trust in science. The high affinity toward scientific research in the current sample is consistent with the recruitment method via the noncommercial online panel SoSci Survey [47], which primarily attracts individuals who regularly participate in online studies without monetary incentives. The high affinity toward scientific research in the current sample could have also led to reactance as a reaction to the science-critical video. Previous research has shown that individuals tend to defend personally held beliefs when confronted with opposing views, sometimes resulting in a reinforcement of those beliefs [66]. We can only speculate that this may, at least partially, explain the observed pattern of increased trust in science following exposure to the science rejection video. It is, however, unlikely that individuals with a low or average affinity toward scientific research would react with a similar positive effect as the participants in our sample. Instead, a general population sample, with a more diverse range of attitudes and affinities, would likely exhibit more varied responses, including indifference or even agreement with the critique, rather than the observed increase in trust toward scientists. Whereas individuals who openly reject science might be difficult to reach for participation in scientific research, the general population likely includes a sufficient number of individuals with moderate or low levels of science skepticism who may be willing to participate in follow-up studies.

Of note, participants of this study who were exposed to the science critical video also reported lower interest in scientific research than those in the control group, despite their increased trust in scientists. A possible explanation for this finding might be that individuals with a high affinity for scientific research react with exhaustion or fatigue when confronted (again) with doubts and debates about the validity of scientific research in the video. This may have resulted in participants distancing themselves from the topic debated in the video and thus reporting lower interest in scientific research.

### Limitations

In addition to the lack of representativeness of the sample discussed above, this study has some additional limitations. First, several measures were either single-item measures or the items had low internal consistency. For trust in scientists, internal consistency improved to an acceptable level (ie, α≥.70) when items with low coefficients were excluded. However, the measure of attitudes toward scientific research was omitted from statistical analysis due to its lack of internal consistency. Additionally, our design only tested a limited number of response strategies. Other response strategies may have been more effective in mitigating the effects of the science-critical video. Furthermore, we tested the impact of a single exposure to a science rejection video of an influencer who was unknown to most of our participants. Social media influencers and their audience are often engaged in a more long-term mediated relationship, involving exposure to different messages over a prolonged period [67]. Thus, any inferences about the impact of science-critical influencers in different contexts should be made with caution. A multiwave examination or investigation of a well-known influencer was beyond the scope of this study, but this should be addressed in future work. Another limitation of this study is the use of exploratory, nondirectional hypotheses. While this approach allowed for an open exploration of potential effects and was consistent with the preregistration of the study, it reflects the current lack of specific theories and theoretical concepts in this particular area of research. Future research could benefit from more theoretically driven, directional hypotheses to increase specificity and strengthen the interpretability of the findings. There may also be several factors that might help to explain or identify individuals for whom science rejection videos are particularly influential, but were not assessed in this study. These potentially relevant factors should be included in future research. Another limitation of the study was that no control group was included that received no stimulus (ie, no video) at all. Furthermore, there was a lack of full comparability between the 2 videos, which differed not only in content (ie, rejection of science vs unrelated to science), but also in influencer identity, filming environment, and production quality (eg, lighting, sound, etc). These differences may have introduced unintended confounding, limiting the internal validity of our findings. Nevertheless, it is a relevant aspect of our design that we provided participants with a control stimulus specifically chosen to have no connection to science rejection in the influencer content and that offered as neutral a setting as possible. Still, future research is needed to address these limitations by using videos from the same influencer, filmed under similar conditions, and by including another control group that receives no stimulus at all, in order to isolate the specific effect of the video content. Finally, the experimental conditions with user comments not only contained opposing comments about the video, but also an equal number of supporting comments. This approach may have increased the authenticity of the response strategies, but it may have also eliminated, mitigated, or at least reduced the impact of negative user comments. Future research should investigate the effect of balance in the comments in order to gain insights into whether a perceived majority of opinion might play a role in the effectiveness of user comments.

### Conclusions

Portrayals of science rejection by an influencer in YouTube videos had relatively little effect on attitudes toward science and scientists of users with a high affinity for scientific research. There was, however, some limited evidence that science rejection videos could potentially simultaneously increase trust in scientists and reduce interest in scientific research in this population. Further research on this effect is needed in order to replicate and verify this finding. Furthermore, user comments that predominantly used factual information in order to critically address and challenge the content of the video tended to be viewed as more informative and trustworthy than other comments; however, the effects of the science rejection video did not vary with different types of user comments. The ability to alter the impact of the science rejection video was somewhat limited for all response strategies tested in the current study, highlighting the need for further research, particularly among individuals with little or no affinity toward scientific research. Future research needs to focus on the development of effective approaches for science communication and social media users to counter false claims by science rejecters who spread public misinformation.

## Appendix

Multimedia Appendix 1 CONSORT 2025 checklist.

Multimedia Appendix 2 Overview of the results of the exploratory analysis.

## Abbreviations

CONSORT: Consolidated Standards of Reporting Trials
GDP: gross domestic product

## Data Availability

Data is available from the corresponding author upon reasonable request.
