# Supplementary material for: Effects of a Video of Science Rejection by a Social Media Influencer and User Comments: Randomized Controlled Trial
Source: J Med Internet Res. 2025 Nov 17;27:e79917. doi: 10.2196/79917 (PMC12670042; doi:10.2196/79917)
Supplement: Multimedia Appendix 2 [file jmir_v27i1e79917_app2.docx]

Supplementary Table 1. *Overview of Descriptive Statistics of Sociodemographic Variables for All Intervention Groups Combined (Groups 1-4) vs. the Control Group in the Online RCT (n = 470)*

| **Variable** | **Control Group**  **(*n* = 94)** | **Intervention Groups 1-4 (*n* =376)** | ***p*** |
| --- | --- | --- | --- |
| *Gender:*  Females *n* (%) | 48 (51.1) | 213 (56.6) | .354^a^ |
| Males *n* (%) | 43 (45.7) | 156 (41.5) | .485^a^ |
| Other *n* (%) | 3 (3.2) | 4 (1.1) | .147^a^ |
| Undisclosed *n* (%) | 0 (0.0) | 3 (0.8) | 1.00^a^ |
| Age *M* (*SD*) | 51.1 (15.0) | 48.6 (16.0) | .183^b^ |
| *Education:*  College/University *n* (%) | 54 (57.4) | 188 (50.0) | .206^a^ |
| High school *n* (%) | 25 (26.6) | 111 (29.5) | .613^a^ |
| Below high school *n* (%) | 15 (16.0) | 77 (20.5) | .384^a^ |

Frequencies (*n*) and percentages (%) or means (*M*) and standard deviations (*SD*) are provided for each group, along with *p* values from independent samples t-tests or Fisher's exact tests assessing group differences.

^a^Fisher’s exact test result

^b^Independent samples t-test result, *t* = 0.88, *df* = 468

Supplementary Table 2. *Overview of Descriptive and Inferential Comparisons of All Outcomes in the Exploratory Secondary Data Analysis between the Combined Science Rejection Video Groups (1–4) and the Control Group in this Online RCT*

|  | **Control group** | **Science Rejection Video (all groups 1-4)** |  |  |
| --- | --- | --- | --- | --- |
| **Outcomes** | Mean score (95% CI) | Mean score (95% CI) | *t* (*d*f)^a^ | *d* (95% CI) |
| Trust in scientists (*n* = 432) | 3.57 (3.36 – 3.77) | 3.80 (3.71 – 3.89) | **2.24 (430)^*^** | **0.27 (0.03 – 0.51)** |
| Science support (*n* = 431) | 76.96 (72.93 – 81.00) | 76.19 (74.44 – 77.95) | -0.37 (429) | -0.05 (-0.28 – 0.19) |
| Interest in scientific research (*n* = 431) | 84.00 (80.56 – 87.44) | 79.56 (77.68 – 81.44) | **-2.09 (429)^*^** | **-0.25 (-0.49** – **-0.02)** |
| Belief in science (*n* = 431) | 3.14 (3.03 – 3.26) | 3.20 (3.14 – 3.25) | 0.82 (429) | 0.10 (-0.14 – 0.34) |
| Conspiracy beliefs (*n* = 431) | 2.98 (2.72 – 3.24) | 2.83 (2.69 – 2.98) | -0.87 (429) | -0.11 (-0.35 – 0.13) |
| Evaluation of video and influencer: |  |  |  |  |
| How much did you like the video? (*n* = 432) | 3.02 (2.78 – 3.27) | 1.48 (1.39 – 1.57) | **-11.68 (106)^***^** | **-1.70 (-1.96** – **-1.43)** |
| How informative was the video? (*n* = 432) | 3.49 (3.26 – 3.72) | 1.45 (1.36 – 1.54) | **-16.57 (111)^***^** | **-2.28 (-2.56** – **-1.99)** |
| How much did you like the influencer? (*n* = 432) | 3.69 (3.46 – 3.92) | 1.97 (1.86 – 2.07) | **-13.67 (430)^***^** | **-1.66 (-1.92** – **-1.40)** |
| How trustworthy was the influencer? (*n* = 432) | 3.98 (3.76 – 4.19) | 1.48 (1.39 – 1.57) | **-23.41 (430)^***^** | **-2.85 (-3.15** – **-2.54)** |

^a*^ *p* < .05. ^**^ *p* < .01. ^***^ *p* < .001. (two-tailed), mean differences with significant *p* values (<0.05) are in bold.
